# Melanocortin-4 Receptor Antagonism Inhibits Colorectal and Anaplastic Thyroid Cancer In Vitro and In Vivo

**DOI:** 10.3390/jcm14041165

**Published:** 2025-02-11

**Authors:** Arianna Bandini, Marta Banchi, Paola Orlandi, Francesca Vaglini, Greta Alì, Gabriella Fontanini, Alessandra Ottani, Daniela Giuliani, Eleonora Vandini, Giulio Francia, Marco Carli, Marco Scarselli, Guido Bocci

**Affiliations:** 1 Dipartimento di Ricerca Traslazionale e delle Nuove Tecnologie in Medicina e Chirurgia, Università di Pisa, 56126 Pisa, Italy; marta.banchi@phd.unipi.it (M.B.); francesca.vaglini@unipi.it (F.V.); marco.carli@phd.unipi.it (M.C.); marco.scarselli@unipi.it (M.S.); 2 Dipartimento di Medicina Clinica e Sperimentale, Università di Pisa, 56126 Pisa, Italy; paola.orlandi@unipi.it; 3 Dipartimento di Patologia Chirurgica, Medica, Molecolare e dell’Area Critica, Università di Pisa, 56126 Pisa, Italy; greta.ali@unipi.it (G.A.); gabriella.fontanini@unipi.it (G.F.); 4 Dipartimento di Scienze Biomediche, Metaboliche e Neuroscienze, Sezione di Farmacologia e Medicina Molecolare, Università di Modena e Reggio Emilia, 41121 Modena, Italy; alessandra.ottani@unimore.it (A.O.); daniela.giuliani@unimore.it (D.G.); eleonora.vandini@unimore.it (E.V.); 5 Border Biomedical Research Center, University of Texas at El Paso (UTEP), El Paso, TX 79968, USA; gfrancia@utep.edu

**Keywords:** MC4R, colorectal adenocarcinoma, anaplastic thyroid cancer, ML00253764, vinorelbine, SN-38, in vitro model, in vivo model

## Abstract

**Background/Objectives** MC4R expression and its role in colorectal and anaplastic thyroid cancers, where resistance to therapy and lack of standard treatments remain significant challenges, are poorly understood. This study aimed to investigate MC4R as a potential therapeutic target in these cancers using the selective antagonist ML00253764 (ML), alone and in combination with vinorelbine (VNR) and irinotecan (or its active metabolite SN-38). **Methods:** Human colorectal adenocarcinoma HT-29, Caco-2, and anaplastic thyroid carcinoma 8305C cell lines were used. MC4R expression was assessed by Real-Time PCR with validated primers (Assay ID Hs00271877_s1), immunofluorescence, and Western blotting. Proliferation and apoptosis assays were conducted with ML, and synergy with VNR and SN-38 was evaluated by Combination Index and Loewe methods. ERK1/2 phosphorylation was measured using an ELISA assay. In vivo studies were conducted by injecting tumor cells into Athymic Nude-Foxn1nu mice, treated with ML, VNR, irinotecan, or their combinations. **Results:** MC4R expression was confirmed in all cell lines. ML treatment inhibited MC4R, producing antiproliferative and pro-apoptotic effects, with IC_50_ values of 7667 ± 2144.6 nM (8305C), 806.4 ± 321.8 nM (HT-29), and 2993 ± 1135.2 nM (Caco-2). In combination with VNR and SN-38, ML exhibited significant synergy in vitro and reduced tumor volume in vivo without causing weight loss or adverse effects in mice. **Conclusions** This study identifies ML as a promising therapeutic agent that, when combined with chemotherapy, may offer a novel strategy for treating colorectal and anaplastic thyroid cancers.

## 1. Introduction

Colorectal cancer is one of the most prevalent malignancies worldwide and a leading cause of cancer-related mortality [1,2]. Despite significant progress in screening methods and the development of treatment approaches such as surgery, chemotherapy, and targeted therapies, effective options for advanced-stage colon cancer remain limited and unsatisfactory [3]. Moreover, a large number of patients develop resistance to conventional therapies, emphasizing the urgent need for novel and more effective treatment strategies that can overcome these limitations [4]. Anaplastic thyroid cancer (ATC) is a rare but extremely aggressive form of thyroid carcinoma, with an incidence of 1–2% among all thyroid cancers [5]. Current treatment modalities, such as radioactive iodine therapy, chemotherapy, radiotherapy, and surgery, have shown limited effectiveness. In the absence of an established standard of care, ATC remains a challenging disease, with poor outcomes and a high mortality rate [6,7].

In both colorectal and anaplastic thyroid cancer, the presence of mutations in the BRAF gene, particularly the V600E mutation, has been shown to significantly worsen prognosis and therapeutic outcomes [8,9,10]. The BRAF V600E mutation is found in approximately 10% of colorectal cancer cases and 25–45% of ATC cases [11,12]. This mutation hyperactivates the mitogen-activated protein kinase (MAPK) pathway, a signaling cascade regulating cell proliferation, differentiation, migration, survival, and angiogenesis [13]. The BRAF V600E mutation increases kinase activity, leading to enhanced phosphorylation of MEK1/2 and activation of ERK1/2, which in turn regulates transcription factors involved in cell growth and survival [10,14]. This resulting dysregulation promotes tumor progression, resistance to apoptosis, and contributes to drug resistance in both cancers [8]. The role of the BRAF mutation in colorectal cancer and ATC underscores its importance as a potential target for therapeutic intervention. In colorectal cancer, the BRAF V600E mutation is associated with chemoresistance, and an overall dismal prognosis [13]. Similarly, in ATC, this mutation contributes to the rapid and invasive growth of the cancer, making it even more resistant to therapies. Despite the development of targeted therapies against BRAF, these options have not fully addressed the need for effective treatments in these tumors, further highlighting the need for innovative therapeutic approaches.

The melanocortin receptor 4 (MC4R) emerged as a promising candidate target for cancer therapy [15,16]. MC4R is a member of the melanocortin receptor family, composed of five receptor subtypes (MC1-5R) [17]. Their ligands that activate the MCRs are melanocortins (α-, β-, and γ- melanocyte-stimulating hormones and adrenocorticotropic hormone), which are peptide hormones generated by proteolytic cleavage of proopiomelanocortin (POMC). Their activity is antagonized by the two endogenous inhibitors, agouti and agouti-related peptide (AgRP) [18,19]. MCRs are all G-protein-coupled transmembrane receptors (GPCRs) associated mainly with stimulatory G protein (Gs) whose activation leads to increased adenylyl cyclase, causing increases in cyclic adenosine monophosphate (cAMP) production, and leading to protein kinase A (PKA) activation [20,21]. However, MC4R has also been found to activate other G proteins, including inhibitory G protein (Gi), and signaling pathways including MAPK, extracellular signal-regulated kinases (ERK) 1 and 2, p38 MAPK, and c-Jun NH2-terminal kinase (JNK) [22]. MC4R is a receptor predominantly expressed in the central nervous system—with a relatively high expression in the thalamus, hypothalamus, and hippocampus—but it is also expressed in the periphery [23].

MC4R is implicated in regulating various physiological processes, including body weight regulation, energy balance, and control of caloric intake [24,25,26,27]. It also plays a crucial role in modulating glucose homeostasis, lipid metabolism, and appetite suppression [28]. MC4R also influences autonomic functions such as thermogenesis, blood pressure regulation, and cardiovascular responses [29]. In addition to metabolic functions, MC4R activity has been linked to behavioral responses, including stress adaptation, highlighting its broad physiological importance in maintaining overall homeostasis [30]. Thus, its role in regulating cell survival and proliferation pathways suggests a potential implication in tumor biology, justifying its selection as a possible target for anticancer treatment.

Vaglini et al. described the presence of the functionally active MC4R in glioblastoma by using the MC4R small-molecule inhibitor ML00253764 (ML) [16]. Furthermore, our research group has recently discovered the expression and important role of MC4R in melanoma human cells [15], but its role in other cancer types has not been explored yet. In particular, there is currently no available information about MC4R expression or activity in anaplastic thyroid cancer or colorectal cancer. ML has been demonstrated as a potent antitumor agent by blocking MC4R signaling pathways against which it exerts selective inverse agonist activity in vitro and in vivo [31]. A potential approach for targeted therapy in cancer cells could involve the deregulation of MC4R signaling pathways and may be able to interfere with important mechanisms involved in cancer initiation, progression, and metastasis. We hypothesized that MC4R inhibition by the melanocortin receptor antagonist ML can reduce tumor proliferation of colorectal cancer cells and of anaplastic thyroid cancer cells and synergize with standard chemotherapy agents. This would greatly enhance the therapeutic effect of available target therapies in patients with these aggressive tumors, thus representing an entirely new treatment that could expand the currently available pharmaceutical options for these two types of tumors. The aim of our research was to examine the presence of MC4R in colorectal and thyroid cancer cells and to evaluate it as a novel target for anticancer therapy in vitro and in vivo by blocking its pathways using the selective antagonist ML in combination with vinorelbine in the case of anaplastic thyroid cancer, and with irinotecan in the case of colorectal adenocarcinoma.

## 2. Materials and Methods

### 2.1. Cell Lines, Materials and Drugs

Human HT-29, Caco-2, and 8305C cell lines were selected based on their molecular characteristics and ability to reproduce clinically relevant colorectal and anaplastic thyroid cancers.

All reagents for cell line cultures were purchased from Sigma Chemical Co. (St Louis, MO, USA). The human colorectal adenocarcinoma HT-29 (HTB-38), BRAF V600E mutated, and Caco-2 wt (HTB-37) were obtained from the American Type Culture Collection (ATCC, Manassas, VA, USA) and they were cultured in ATCC-formulated McCoy’s 5a Medium Modified (Catalog No. M9309) and Eagle’s Minimum Essential Medium (Catalog No. M2279), respectively. The media were supplemented with heat-inactivated fetal bovine serum (FBS) (Catalog No. F7524) at 10%, 1% Sodium Pyruvate, 1% L-glutamine (Catalog No. G7513), and 100 U/mL penicillin, 100 mg/mL streptomycin (Catalog No. P4333). The Human ATC cell line 8305C with BRAF V600E mutation (ACC 133) was bought from the Leibniz Institute DSMZ-German Collection of Microorganisms and Cell Cultures GmbH (DSMZ, Germany). The 8305C cell line was cultured in RPMI-1640 medium (Catalog No. R8758) completed with 2 mM l-glutamine and 20% of FBS. The passage number of cell lines was indicated by the sellers. Cells were maintained at 37 °C in 5% CO_2_ and 95% humidity. Human umbilical vascular endothelial cells (HUVECs) (PCS-100-013) were acquired from the American Type Culture Collection (Manassas, VA); they were routinely grown in 75-cm^2^ tissue culture flasks and maintained in MCDB131 culture medium (Catalog No. M8537) supplemented with 10% heat-inactivated FBS, L-glutamine (2 mM), heparin (10 U/mL), EGF (10 ng/mL), and basic fibroblast growth factor (5 ng/mL), and kept in a humidified atmosphere of 5% CO_2_ at 37 °C. Sterile cell culture plastics were obtained from Sarstedt (Nümbrecht, Germany). Vinorelbine (Catalog No. S4269), Irinotecan (Catalog No. S5026), and SN-38 (Catalog No. S4908), the active metabolite of irinotecan used in vitro, were acquired from Selleckchem (DBA Italia, Milan, Italy), and they were solubilized in dimethyl sulfoxide (DMSO). The ML00253764 compound (Cat. No. 4854/10) (chemical name: 2-[2-[2-(5-Bromo-2-methoxyphenyl) ethyl]-3-fluorophenyl]-4,5-dihydro-2-1H-imidazole hydrochloride), a small-molecule (non-peptide) selective MC4R antagonist was obtained from Tocris Bioscience (Bristol, UK), and was solubilized in sterile water. To conduct in vitro experiments, all drugs were dissolved to a 10 mM stock solution. The concentration of DMSO in the negative control media was the same as that used to produce the highest concentration of each drug in growth media for the same experiment.

### 2.2. MC4R Gene Expression Analysis by Real-TimePCR, Immunofluorescence and Western Blotting

To examine the expression of the MC4R gene in the HT-29, Caco-2 and 8305C cell lines, the cells were plated to a 72 h replication cycle. The RNase Mini Kit (Qiagen, Valencia, CA, USA) was used to extract RNA from tumor cells. Reverse-transcribed RNA (1 μg) as previously described [10] yielding cDNA that was diluted (2:3) and amplified by QRT-PCR using the Applied Biosystems 7900HT (Applied Biosystems, Carlsbad, CA, USA) sequence detection system in order to quantify the MC4R mRNA. MC4R-validated primers (Assay ID Hs00271877_s1) were purchased from Applied Biosystems. The manufacturer’s recommendations for primer concentration optimization and PCR heat cycling settings were followed. Glyceraldehyde 3-phosphate dehydrogenase (GAPDH) (Catalog No. 4485713) was used as a reference for amplifications, and fold changes relative to GAPDH expression were used to quantify gene expression. Every experiment was repeated, independently, three times with at least nine samples.

In order to evaluate the expression of the MC4-receptor density in HT-29 and 8305C cells by immunofluorescence, the cells were first fixed onto slides and subsequently treated with a secondary goat anti-rabbit fluorescent antibody (diluted 1:250 in PBS; Abcam; Cambridge, MA, USA). Trimethyl-[3-[4-[(E,3Z)-3-(3-methyl-1,3-benzothiazol-2-ylidene)prop-1-enyl]quinolin-1-ium-1-yl]propyl]azanium was used to stain cells. A confocal Leica TCS SP5 laser-scanning microscope was used to view the coverslips after nuclear staining with astanium diiodide (TO-PRO3; Molecular Probes; Eugene, OR, USA) diluted 1:1000 in PBS (Catalog No. D8537). We used magnifications of both × 40 and × 60.

To perform Western Blotting HUVEC, colorectal tumor cells (HT-29 and Caco-2), were lysed in RIPA cold lysis buffer containing a combination of protease inhibitors and then incubated on ice for 15 min, after which they were centrifuged at 14,000× *g* for 15 min at 4 °C. Surfactants were gathered, and Bradford’s method was used to determine the protein content. Proteins were denatured in reduction buffer (40 μg per sample), separated by SDS-polyacrylamide gel electrophoresis (PAGE), and transferred to PVDF (polyvinylidene fluoride) membranes. Then, membranes were blocked in 5% non-fat milk for 1 h at a temperature of 25 °C, washed, and incubated overnight at 4 °C with a primary antibody for MC4R (dilution 1:500, ab75506; Abcam). The day after, membranes were washed and then treated for 1 h at room temperature with a peroxidase-conjugated secondary antibody (Cell Signaling Technology; Danvers, MA, USA). Following washing, bands were detected using the enhanced chemiluminescence Luminata Forte Western RP Substrate (Millipore, Billerica, MA, USA) on a Licor station. Using an anti-β-tubulin antibody (Cell Signaling), the blots were examined for β-tubulin expression in order to demonstrate equivalent loading.

### 2.3. Antiproliferative and Apoptosis Assays

The in vitro antiproliferative activity was examined for the HT-29, Caco-2, and 8305C cell lines. The experimental protocols have been reported before with some changes [10]. In summary, ML (0.001–50 μM) was administered alone and in combination with SN-38 or vinorelbine (0.001–100 μM) to treat cells continuously for 72 h, by adding new drug aliquots with fresh media every 24 h. Twenty-four-well sterile plastic plates were used to seed the tumor cells. Using a hemocytometer, viable cells (determined by trypan blue dye exclusion) were counted. The data are reported as the percentage of the vehicle-treated cells. Every experiment was conducted, separately, three times with at least three samples for every drug concentration. A non-linear regression fit of the mean values of the data obtained in triplicate experiments (at least nine wells for each drug concentration) was used to estimate the drug concentration that resulted in a 50% reduction of cell growth (IC_50_) compared to vehicle-treated samples.

HT-29 and 8305C 5 × 10^5^ cells were seeded in 100-mm sterile dishes, and they were incubated for 72 h with ML, vinorelbine, SN-38, and their concomitant combination, at their respective antiproliferative IC_50_s, and with vehicle alone in order to determine apoptosis. They were collected at the end of the treatment period, and samples were examined using the Cell Death Detection enzyme-linked immunosorbent assay (ELISA) Plus kit (Roche, Basel, Switzerland). The data are presented as percentages in relation to the vehicle-treated absorbance mean value, which was set at 100% and derived from a minimum of nine samples. Every experiment was repeated three times separately, using a minimum of three samples to determine the drug concentrations.

### 2.4. In Vitro Determination of Synergism Between ML Plus Vinorelbine or SN-38 in Tumor Cells

The activity of combining ML plus vinorelbine, or SN-38, was investigated in 8305C and HT-29 cells using a concomitant program of an established molar concentration ratio of 1:1, including ML (0.01–50 μM) plus vinorelbine or SN-38 (0.01–50 μM) for 72 h. The Loewe additivity model and the combination index (CI) approach were used to determine the possible synergistic activity between ML and vinorelbine or SN-38 [19]. Briefly, a multiple drug-effect equation was used to evaluate the synergism or antagonism between ML and vinorelbine or SN-38. The results were quantified by the CI, where CI < 1, CI = 1, and CI > 1 mean synergism, additive effect, and antagonism, respectively.

Using the standard isobologram for mutually exclusive effects, the CI value was evaluated as follows in order to determine the kind of interaction (synergistic, additive, or antagonistic) between ML and vinorelbine or SN-38: CI = [(D)1/(DX) 1] + [(D)2/(DX)2], where (D)_1_ and (D)_2_ are the concentrations of ML and vinorelbine, or SN-38, that combined block cell proliferation to the equivalent percentage, and (Dx)_1_ and (Dx)_2_ are the concentrations of ML and vinorelbine or SN-38, respectively, that cause a specific percentage of inhibition of cell proliferation.

The theoretical concentration decrease that could be reached for each drug in the combination compared to the concentration of each drug alone that would produce the equivalent effect is represented by the dose reduction index (DRI).

(DRI)_1_ = (DX)_1_ /(D)_1_ and (DRI)_2_ = (DX)_2_/(D)_2_

The CI and DRI indexes were calculated with the CalcuSyn v.2.0 software (Biosoft, Cambridge, UK). The synergistic, additive, and antagonistic effects of the combined drugs were also validated and graphically summarized using the Loewe additivity model, with the Combenefit software (v.2.021), an interactive platform for the analysis and visualization of drug combinations [20].

### 2.5. ERK1/2 (pTp 185/187) ELISA Assay

Treatment of HT-29 and 8305C (5 × 10^4^ cells/well) with ML00253764 or vehicle alone lasted for 72 h. At the conclusion of the experiment, the cells were harvested and quickly frozen in liquid nitrogen in order to determine pERK1/2 levels. Following the reported protocol for cell lysis [10], the amount of total protein was quantified. Then, using the PhosphoDetect^®^ ERK1/2 (pThr185/pTyr187) ELISA Kit (Calbiochem, Merck Millipore, MO, USA), an equivalent number of total proteins was assayed for human ERK1/2 phosphorylation in each sample. The results were normalized by total protein ERK1/2 concentration, which was determined by the ERK1/2 ELISA kits, respectively. Using the Multiskan Spectrum (Thermo Labsystems, Milan, Italy) microplate reader with the setting set at 450 nm, the relative optical density was measured.

### 2.6. In Vivo Experiments

Athymic Nude-Foxn^1nu^ male mice (20–25 g) were purchased from Envigo (Milan, Italy) and kept in microisolator cages with ventilated racks. The animals were handled aseptically, and they were provided with unrestricted access to sterile food and water. All animal procedures and housing were conducted in accordance with the approval of the Academic Organization Responsible for Animal Welfare (OPBA, Organismo Preposto per il Benessere Animale) of the University of Pisa and of the Italian Ministry of Health (authorization number 191/2019-PR). The previously reported in vivo experimental protocol was used with some adjustments [15]. Briefly, on day 0 of the experiment, 3 × 10^6^ ± 5% viable HT-29 and 8303C cells/mouse in 0.2 mL serum-free medium were injected subcutaneously (s.c.) in each mouse between the scapulae. Every two to three days, the weights of the mice were recorded, and a caliper was used to measure the tumor volume in two crosswise lines. The tumor volume (mm^3^) was measured as [(w_1_ × w_1_ × w_2_) × (π/6)], where w_1_ and w_2_ were the largest and the smallest mass widths (mm), respectively. In our study, we adhered to the principles of the 3Rs (Replacement, Reduction, and Refinement) to ensure animal welfare [32]. Each experiment involved the minimum number of mice necessary to obtain statistically significant results. Mice were randomly assigned to the different groups of six animals just before the beginning of the treatment using a random number generator. Mice were treated as follows: ML 30 mg/kg s.c. daily, vinorelbine 10 mg/kg i.p. once a week, CPT-11 100 mg/kg i.p. once a week, and their concurrent combination (ML+ vinorelbine: 30 mg/kg + 10 mg/kg or ML+CPT-11: 30 mg/kg + 100 mg/kg) were administered to the mice following the development of an established tumor (at an approximate size of 50 mm^3^). All compounds were prepared fresh and stored according to the manufacturers’ guidelines to guarantee stability and integrity during use. Doses of each drug used in all experiments were selected based on previous studies [15,33,34,35]. At the end of the experiment, mice were sacrificed by an overdose of anesthetic. Tumors were then excised and measured. In addition, animals were euthanized with anesthesia if they lost 20% of their body weight during the experiment to minimize suffering.

### 2.7. Immunohistochemistry

Immunohistochemistry (IHC) was performed as previously described [15]. Briefly, tumor tissue samples were fixed in 10% neutral-buffered formalin for 12–24 h and embedded in paraffin. Sections of the tumor (5 μm thick) were stained with hematoxylin and eosin. Immunostaining was carried out with a Benchmark immunostainer (Ventana, Tucson, AZ, USA) and using the avidin-biotin-peroxidase complex (ABC) method and counterstained with hematoxylin. Negative controls were performed by omitting the primary antibodies. IHC was evaluated independently by two pathologists. The microvessel number was established using an anti-CD31 polyclonal antibody (clone JC70; cat. 760–4378, Ventana Medical System, Roche Diagnostic, Tucson, Arizona, USA.). The identification of apoptosis was performed using a rabbit polyclonal antibody that recognizes active caspase-3 (diluted 1:2000; cat. ab2302, Abcam, Cambridge, UK).

### 2.8. Statistical Analysis

For the data analysis, investigators were blinded to which samples/animals represented treatments and controls. The data (mean ± SD or SEM) underwent to analysis of variance (ANOVA), followed by the Student–Newman–Keuls test. The level of significance was established at *p* < 0.05. Analyses were carried out with the GraphPad Prism software package version 7.0 (GraphPad Software, Inc., San Diego, CA, USA). For the in vivo experiments, 6 animals were used per group in order to give 80% power to one-way ANOVA analysis, against a difference in tumor volumes equal to or greater than 0.50*sd, with a nominal alpha error rate = 0.05. The power calculation was carried out using G*Power 3 software version 3.1.9.7.

## 3. Results

### 3.1. In Vitro Experiments

#### 3.1.1. MC4R Expression in HT-29, Caco-2 and 8305C Cell Lines

**Figure 1:**
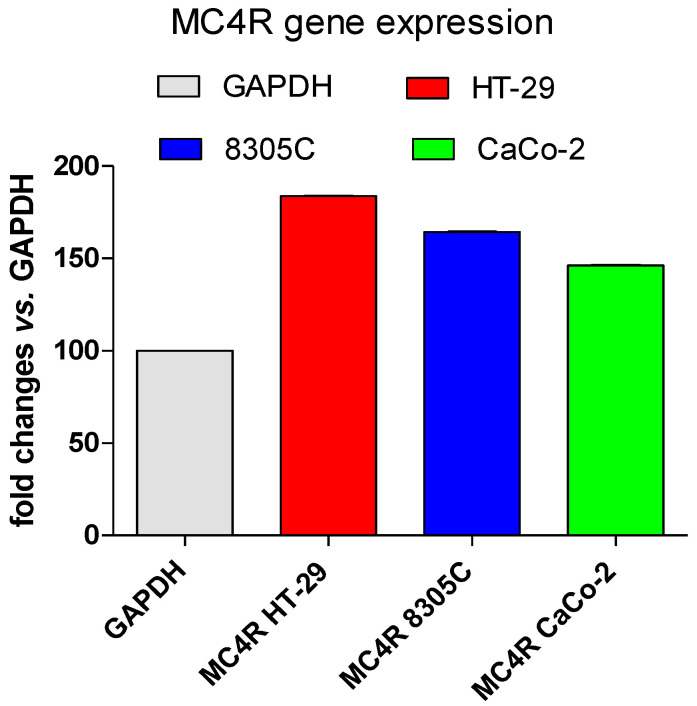
Melanocortin receptor-4 (MC4R) gene expression in HT-29, 8305C, and Caco-2 cell lines assessed by Real-Time PCR analysis; glyceraldehyde 3-phosphate dehydrogenase (GAPDH); the results are shown as fold changes relative to GAPDH expression.

**Figure 2:**
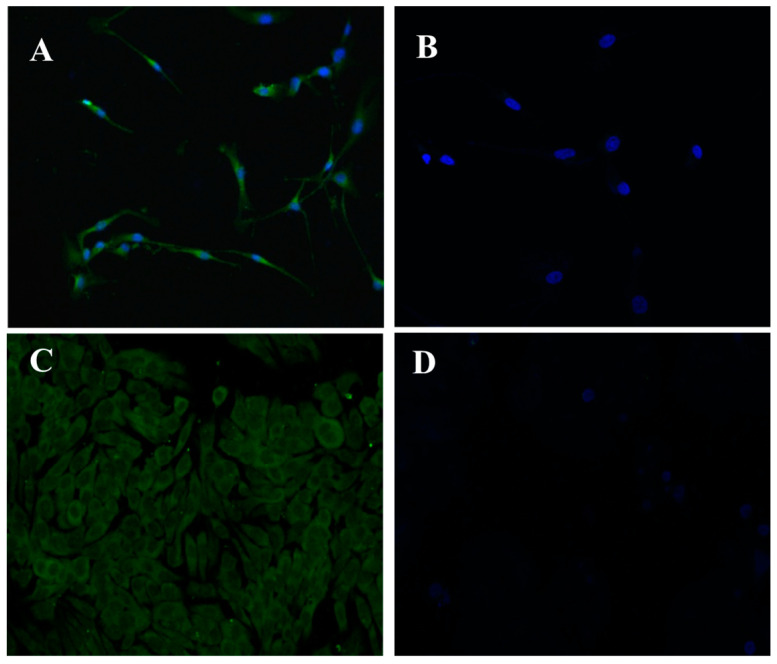
Immunofluorescence of 8305C cells stained with MC4R-antibody (**A**) and negative control (**B**), performed by omission of the primary antibody; immunofluorescence of HT-29 cells stained with MC4R-antibody (**C**) and negative control (**D**).

**Figure 3:**
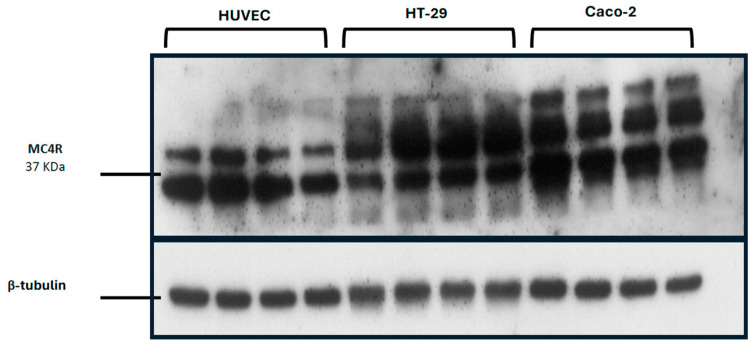
Immunoblotting of MC4R protein from total cellular lysates of HT-29 and Caco-2 cell lines and endothelial cells (HUVEC). The western blot bands correspond to MC4R and β-tubulin.

Real-time PCR analysis was performed with HT-29, 8305C, and Caco-2 cell lines, and it revealed the presence of MC4R mRNA in all three cell lines. Particularly, as shown in Figure 1, the MC4R gene shows readily detectable levels of expression, and these were normalized to glyceraldehyde 3-phosphate dehydrogenase (GAPDH) expression levels. It was observable that 8305C thyroid cancer cells were positive for MC4R protein (Figure 2A) whereas in the absence of the antibody, they were negatively stained (Figure 2B). Moreover, HT-29 colorectal cancer cells were also positive for the MC4R protein (Figure 2C) when comparing the immunofluorescence of proliferating cells to negative controls in the absence of the primary antibody anti-MC4R (Figure 2D). Western blot analysis confirmed the high concentration of MC4R protein in both HT-29 and Caco-2 cell lines compared to HUVEC non-tumor endothelial cells (Figure 3), with two marked bands clearly visible (the upper one probably represents an intermediate glycosylated MC4R).

#### 3.1.2. Antiproliferative Effect of ML00253764

**Figure 4:**
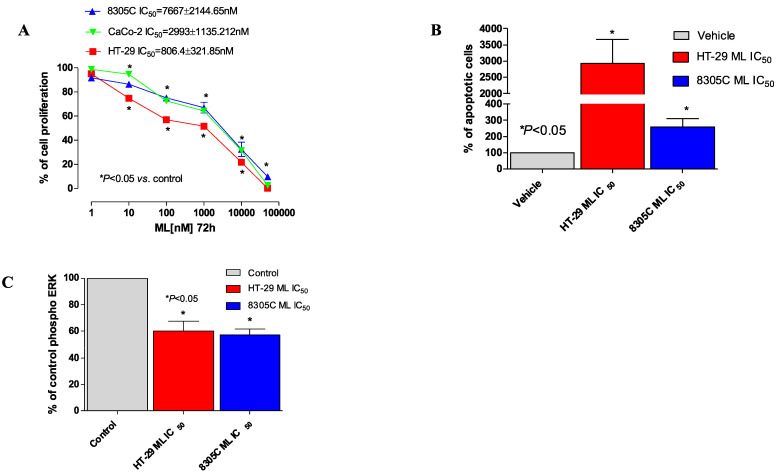
Antiproliferative effects of ML00253764 (ML) in vitro on human 8305C, Caco-2, and HT-29 cell lines (**A**). The antiproliferative effects of the drug were studied after 72 h of exposure. The data are presented as mean (±SD) percentage values of vehicle-treated cell proliferation. * *p* < 0.05 vs. controls. Apoptosis analysis (**B**) and inhibition of ERK1/2 phosphorylation (**C**) in HT-29 and 8305C cell lines treated with ML00253764 (ML) at respective IC_50_s. The data are expressed as percentages versus the mean value of vehicle-treated absorbance (obtained from at least nine samples) that was set at 100%. Columns and bars, mean values ±SEM, respectively. * *p* < 0.05 vs. vehicle-treated controls.

After a 72 h treatment with the MC4R antagonist ML00253764, an antiproliferative effect was observed in 8305C, HT-29, and Caco-2 lines as indicated by the calculated IC_50_s of 7667 ± 2144.6 nM, 806.4 ± 321.8 nM, and 2993 ± 1135.2 nM (Figure 4A), respectively, demonstrating a concentration-dependent pharmacological activity. Figure 4A illustrates that treatment with 100 nM of ML drug resulted in approximately 60 ± 2.19%, 30 ± 2.76%, and 75 ± 0.85% cell survival in HT-29, CaCo-2, and 8305C cells, respectively, compared to the 100% survival observed in the vehicle-only control. As shown in Supplementary Figure S1A, vinorelbine treatment for 72 h decreased the proliferation of 8305C cells, with an IC_50_ of 13.6 ± 7.44 pM. SN-38, the active metabolite CPT-11, used for the in vitro experiments, was also very effective on HT-29 cell growth, showing an IC_50_ value of 81 ± 26 nM after 72 h drug exposure (Supplementary Figure S1B).

#### 3.1.3. Apoptosis Induced by ML00253764

An ELISA test was used to quantify the apoptotic processes. In comparison to cells treated with a vehicle, Figure 4B indicates a significant increase in the amount of DNA fragmentation at the ML IC_50_ after 72 h of treatment in HT-29 cells. Additionally, the ML IC_50_ concentration significantly increased the rate of apoptosis in 8305C cells after 72 h.

#### 3.1.4. Decrease of ERK1/2 Phosphorylation Caused by ML00253764

As shown in Figure 4C, ML decreased the phosphorylation of ERK 1/2 in both HT-29 and 8305C cell lines at concentrations that significantly inhibited 50% of the growth of those cancer cells.

#### 3.1.5. Synergistic Effect of ML00253764 and SN-38 or Vinorelbine on Colon and ATC Cells

**Figure 5:**
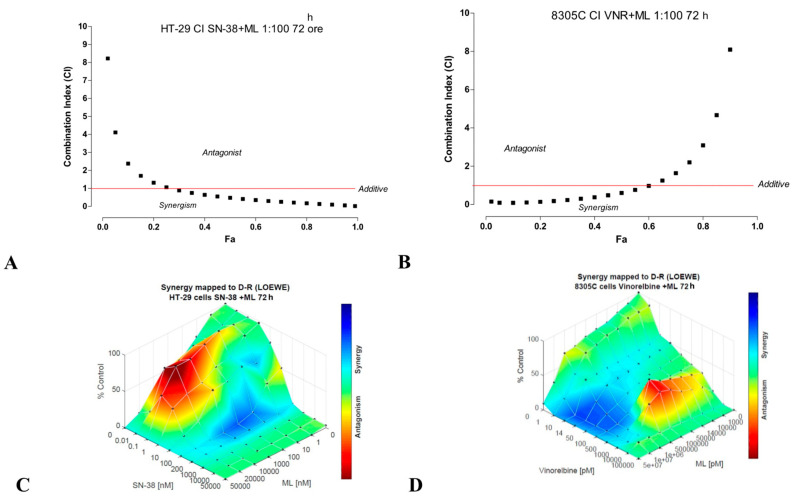
Combination Index (CI)-fraction affected (Fa) plot at 72h concomitant combination treatment of ML+ vinorelbine in HT-29 cells proliferation inhibition. The symbols represent the combination index values (synergism CI < 1) per fraction of cells affected by the combination (**A**). The 3-dimensional landscape of the dose matrix of combination responses for ML and vinorelbine based on the Loewe model in HT-29 (**C**), where blue reflects evidence of synergy and red represents evidence of antagonism. Combination Index (CI)-fraction affected (Fa) plot of 72h concomitant combination of ML+ SN-38 in 8305C cell proliferation inhibition. The symbols represent the combination index values (synergism CI < 1) per fraction of cells affected by the combination (**B**). The 3-dimensional landscape of the dose matrix of combination responses for ML and SN-38 based on the Loewe model in 8305C (**D**), where blue reflects evidence of synergy and red represents evidence of antagonism.

The simultaneous combination of ML00253764 and SN-38 showed a very strong synergistic activity on HT-29 cells from small fractions affected (Fa), with the Combination index value far below 1 (Figure 5A), producing an antiproliferative effect comparable to what would be expected by the simple sum of the effects if the drugs were administered individually. This strong synergy suggests that combining ML00253764 with SN-38 could allow dose reduction while maintaining efficacy, potentially minimizing toxicity and adverse effects.

The simultaneous exposure of 8305C cells to different concentrations of ML00253764 and vinorelbine for 72 h showed marked synergism (CI < 1) for the fraction of affected cells (Fa) up to 60% (Figure 5B). The Loewe additivity model, which can be used to calculate the drug combination effect and map it as a three-dimensional synergy diagram, was also used to validate the results for both cell lines. This showed that the association between SN-38 or vinorelbine plus ML was synergistic in both HT-29 and 8305C (Figure 5C and 5D, respectively) and DRI > 1 (Table 1), supporting the possibility of reducing drug doses while maintaining therapeutic efficacy. Specifically, ML+SN-38, in HT-29, showed a DRI value of up to 668.78 at 90% growth inhibition, while for 8305C cells, ML+VNR has a DRI of 624.56 at the same inhibition level (Table 1). These values indicate that significantly lower doses of each drug could be used while maintaining therapeutic efficacy, thereby reducing systemic toxicity.

#### 3.1.6. Decrease in Tumor Growth In Vivo Observed with the ML Plus Vinorelbine and ML Plus CPT-11 Combination

**Figure 6:**
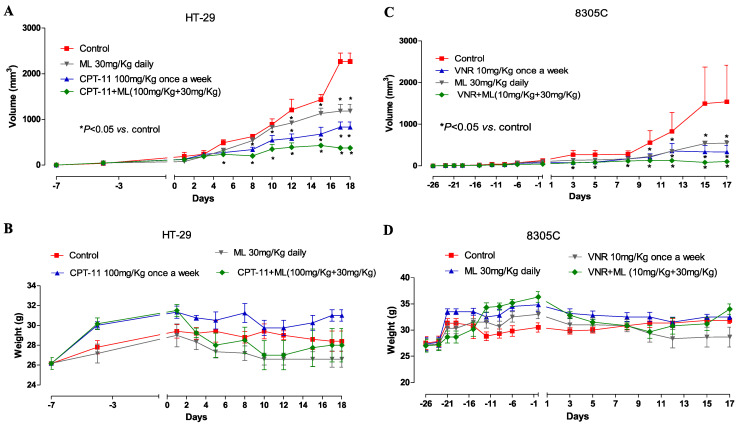
In vivo therapeutic effect (**A**) of ML 30 mg/kg s.c. every day alone, CPT-11 100 mg/kg once a week, and ML concomitant combination with CPT-11 (100 mg/kg + 30 mg/kg) on HT-29 xenotransplants in CD nu/nu mice, and (**C**) of ML 30mg/kg daily alone, VNR 10 mg/kg once a week, and concomitant combination of ML, and VNR (10 mg/kg + 30 mg/kg) on 8305C xenotransplants in CD nu/nu mice. Body weight of HT-29 (**B**) and 8305C (**D**) tumor-bearing control mice and mice treated with ML and vemurafenib alone or in combination. Symbols and bars, mean ± SEM; * *p* < 0.05 vs. controls.

**Figure 7:**
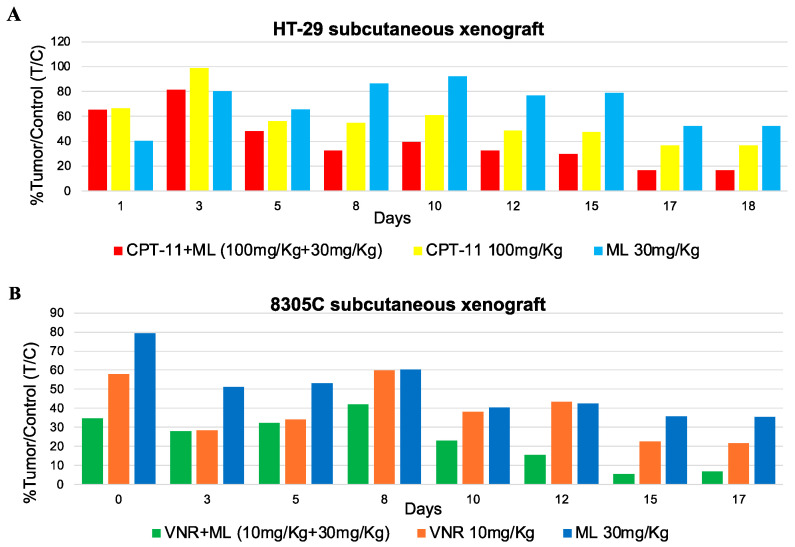
In vivo antitumor effects of single-agent and combination treatments with CPT-11 + ML or VNR + ML on HT-29 (A) and 8305C (B) subcutaneous tumor xenografts in nude mice, expressed as % Tumor/Control (T/C) values.

When injected subcutaneously into male Athymic Nude-Foxn^1nu^ mice, HT-29 and 8305C cells produced tumors after 7 and 26 days, respectively. When compared to mice treated with vehicle alone, both ML (30 mg/kg per day) and CPT-11 (100 mg/kg once a week) monotherapies limited the growth of HT-29 tumors (Figure 6A), and this was observed as early as day 7 into therapy. Notably, as shown in Figure 7A, already after 8 days of treatment with the ML+CPT-11 combination, a decrease in tumor volume of 33% compared with the control could be observed.

Treated mice also showed no significant signs of weight loss (Figure 6B). The simultaneous administration of ML and vinorelbine markedly inhibited the growth of the 8305C tumor in comparison to controls, and the combination was well tolerated (Figure 6C). As illustrated in Figure 7B, already after 8 days of treatment with the ML+VNR combination, tumor volume was reduced by 42% compared to the control group.

The VNR and ML produce the same antineoplastic effect, with notable variations in mean tumor volume between the combination and control groups (Figure 6C). No discontinuation of treatment administration was required in these experiments (Figure 6D), indicating that the combination was well tolerated and did not induce significant toxicity in vivo.

### 3.2. Immunohistochemistry of HT-29 and 8305C Subcutaneous Tumors

**Figure 8:**
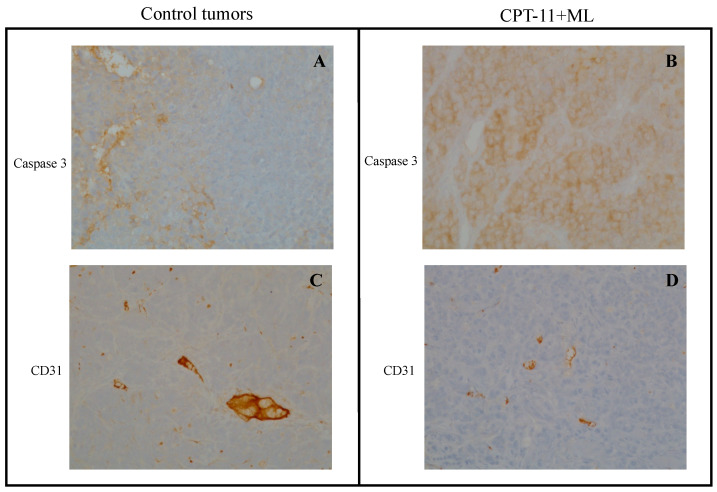
Representative microscopic images of immunohistochemical staining of subcutaneous tumor samples HT-29 treated with saline (control), ML00253764 (ML), and irinotecan (CPT-11) at the end of an 18-day treatment, including active caspase 3 (**A** and **B**, respectively) and CD31 (**C** and **D**, respectively). Magnification, ×20.

**Figure 9:**
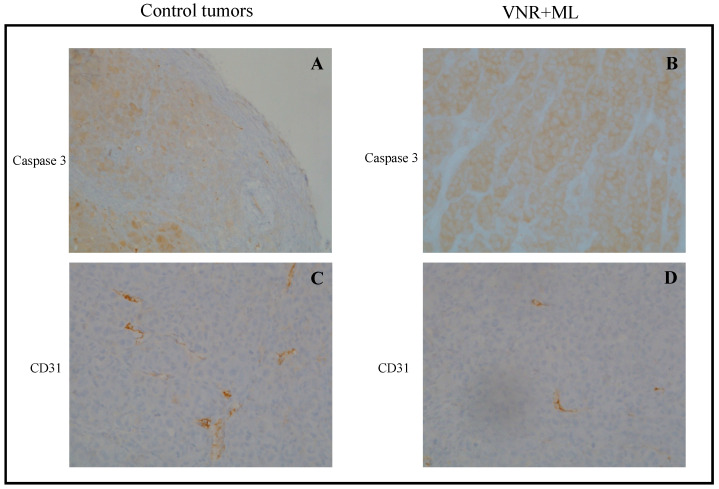
Representative microscopic images of immunohistochemical staining of subcutaneous tumor samples 8305C treated with saline (control), ML00253764 (ML), and vinorelbine (VNR) at the end of a 17-day treatment, including active caspase 3 (**A** and **B**, respectively) and CD31 (**C** and **D**, respectively). Magnification, ×20.

The values for the apoptotic index (reported as caspase 3 expression) and for microvascular density (i.e., CD 31+ positive staining) of vehicle- and drug-treated mice obtained from immunohistochemical analysis of subcutaneous tumor tissue derived from HT-29 and 8305C cell injections are shown in Table 2. These findings support the proposed mechanism of MC4R inhibition, which enhances apoptosis and reduces angiogenesis, contributing to the observed antitumor effects. In all treated samples, there was a statistically significant increase in activated Caspase-3 staining, as well as a decrease in microvascular density (CD31+ expression) at both active concurrent treatments in HT-29 and 8305C tumors (Figure 8 and Figure 9 show representative microscopic images, respectively) compared to control groups. Increased caspase-3 suggests that MC4R inhibition promotes apoptotic pathways, while reduced CD31+ staining indicates altered tumor vasculature, which may limit nutrient supply and tumor progression. Moreover, MC4R, being present on endothelial cells (HUVEC), as demonstrated in our study, could also contribute to reduced microvascularization through its receptor activity. Together, these effects strengthen the therapeutic potential of MC4R inhibition in tumor growth suppression.

## 4. Discussion

The MC4R receptor has been demonstrated to be expressed and to be active in glioblastoma and melanoma cancer cells in two previous studies [9,10]. No additional information about the receptor’s function in other tumor types has yet been released as far as we know. Therefore, the objective of our research was to investigate, for the first time, the role of MC4R in anaplastic thyroid cancer and colorectal cancer, and to determine whether it could represent a novel potential molecular target.

Our data show that MC4R is present and highly expressed in human colorectal adenocarcinoma (wt Caco-2 and *BRAF*-mutated HT-29) and in anaplastic thyroid carcinoma cells (*BRAF*-mutated 8305C). Overexpression of MC4R, as demonstrated by real-time PCR, immunofluorescence, and Western blot analysis, highlights the significant presence of this receptor in human ATC and colorectal tumor cells. This suggests a potential role for MC4R in key pathways that drive tumor growth and survival.

The selection of these cell lines was carried out carefully to ensure a heterogeneous representation of the tumor subtypes commonly found in the population, despite the limited number of models. Caco-2 cells, which are wild-type for BRAF, were chosen because they are well-characterized, differentiated, and representative of the most prevalent form of colorectal cancer. On the contrary, HT-29 cells harbor the BRAF mutation, which is associated with a more aggressive tumor behavior [13]. Similarly, the 8305C cells, derived from anaplastic thyroid carcinoma, also harbor the BRAF mutation, making them a relevant model for the study of aggressive thyroid cancer [11].

The immunofluorescence study in 8305C and HT-29 cells showed a high MC4R positive stain in the plasma membrane of both cell lines, compared to controls. Western blot analysis further validated a marked difference in MC4R protein expression between normal cells, HUVECs, and both ATC and colorectal cancer cells. These findings support the hypothesis that MC4R may contribute to tumor growth and survival mechanisms across different tumor types, potentially interacting with oncogenic pathways, including those driven by BRAF mutations.

We chose to test the small-molecule MC4R selective antagonist ML00253764, originally developed by Vos and colleagues [36], which was previously employed to block tumor-induced weight loss in mice. This selective antagonist has already been used by Nicholson et al. to prevent malignant cachexia in vivo, specifically in a Lewis lung carcinoma model at a dose of 15 mg/kg twice per day and in a colon carcinoma tumor model at a subcutaneous dose of 30 mg/kg [31,36]. To date, no data are available on the antiproliferative and cytotoxic effect of MC4R antagonists in the colon and ATC that could be directly compared with our findings. However, the use of ML, both alone and in combination with chemotherapeutic drugs, exhibited strong synergistic effects in the treatment of *BRAF-mutated* A-2058 and WM 266-4 melanoma cell lines, as well as in U-87 and U-118 glioblastoma cells [15,16]. This new observation regarding the melanocortin receptor 4 adds to the growing body of knowledge about the role of the melanocortin system in cancer biology, which to date has not been explored in colorectal and thyroid cancers. The present in vitro experiments demonstrate that ML00253764 is highly effective at inhibiting cell proliferation at micro and nanomolar concentrations in a concentration-dependent manner across all tested cell lines. The IC_50_ values for ML varied between the three cell lines, with the most potent effect observed in *BRAF-mutated* HT-29 colorectal cancer cells, followed by the *BRAF-mutated* 8305C and then Caco-2 cells. This pattern is similar to our previous observations in melanoma and glioblastoma, where the highest sensitivity to ML was seen in melanoma cells, particularly those with *BRAF* mutations [15,16]. In our melanoma study, the IC_50_ for ML in BRAF-mutated A-2058 cells was 11.1 nM, making it the most responsive cell line to the antagonist. In contrast, glioblastoma cells showed a lower sensitivity, with IC_50_ values in the micromolar range (11.36 µM for U-87 cells and 6.56 µM for U-118 cells). Although ML was most effective in melanoma, as shown by this study, it still exhibited a significant inhibitory effect in colorectal cancer and ATC cells. In particular, the IC_50_ values for the 8305C, HT-29, and Caco-2 cell lines were 7.667 nM, 2144.6 nM, and 806.4 nM, respectively. These findings suggest that while BRAF-mutated melanoma cells exhibit the highest sensitivity to ML00253764, the compound also exerts notable inhibitory effects in colorectal and anaplastic thyroid cancer models, reinforcing its potential as a therapeutic agent for multiple tumor types. Furthermore, these results suggest variable responses to MC4R inhibition, potentially associated with differences in downstream signaling pathways or receptor expression levels in each tumor population. The ability of MC4R antagonists to both stop proliferation and cause cell death is highlighted by the induction of apoptosis, as seen by increased DNA fragmentation in treated cells induced by ML at IC_50_ concentration, compared to controls, for both HT-29 and 8305C cell lines. As previously noted in glioblastoma and melanoma cells, pro-survival pathways in these tumor types are activated in part by MC4R signaling, and its pharmaceutical anticancer efficacy is correlated with the activation of ERK1/2 [15,16]. This strengthens the idea that MC4R inhibition is particularly effective in tumor types that rely on ERK1/2 signaling for survival, a pathway highly activated in BRAF-mutated cancers.

The MC4R, like other melanocortin receptors, is coupled to transmembrane G proteins, primarily activating the stimulatory G protein and adenylate cyclase as a conventional signaling pathway [23,24]. The MC4R not only activates the Gs-cAMP-PKA pathway but also engages additional signaling pathways involving other G proteins, including Gαi/Gαo, Gαq, and the activation of mitogen-activated proteins/extracellular signal-regulated kinases (MAPK/ERK)-dependent G proteins [20,37].

Among these, it has been hypothesized that the phosphorylation of ERK 1/2 is critical for MC4R activity both in vivo and in vitro, although the physiological function of this pathway remains unexplored [38,39,40]. In particular, studies in heterologous expression systems have shown that in vitro MC4R agonist stimulation activates ERK 1/2, suggesting that MC4R does not directly activate the ERK cascade but rather that G proteins coupled to MC4R are necessary for ERK activation [41]. One proposed mechanism linking MC4R activation with ERK1/2 phosphorylation involves the suppression of AMP-activated protein kinase (AMPK) after α-MSH stimulation and the subsequent activation of PKA via Gs, which leads to ERK phosphorylation [27,42]. This pathway has long been implicated in the promotion of cancer cell proliferation and survival, which is why the inhibition of ERK1/2 phosphorylation is considered a critical step in reducing tumorigenesis. Blockade of the MC4R receptor by its endogenous antagonist AgRP and the three small-molecule reverse agonists (Ipsen 5i, ML00253764, and MCL0020) has been shown to reduce basal signaling in the cAMP pathway and to activate ERK1/2 signaling [32]. Notably, the application of SHU9119, a partial agonist of MC5R and antagonist of MC3/4 receptors, suppressed NDP-MSH-mediated ERK1/2 activation in GT1-1 cells endogenously expressing MC4R, or in Chinese hamster ovary (CHO)-K1 cells stably transfected with MC4R [33,34]. In our study, ML administration in vitro also blocked the ERK 1/2 pathway in a time- and concentration-dependent manner in A-2058 melanoma cells, whereas the inhibition of ERK 1/2 phosphorylation completely disappeared in A-2058 cells with no MC4R expression [9]. These findings suggest that the pharmacological antitumor activity of MC4R inhibition is strongly associated with the suppression of ERK1/2 phosphorylation, as we have already also observed in glioblastoma cells previously [16]. Furthermore, while ERK1/2 inhibition clearly plays a significant role in the observed antiproliferative and pro-apoptotic effects of MC4R inhibition, it is important to acknowledge that ERK1/2 is likely only one of several key signaling pathways involved [43]. Other pathways, such as the PI3K/Akt pathway, may also contribute to the overall effects of MC4R inhibition. The PI3K/Akt pathway, which is frequently activated in various cancers, including glioblastoma, where it plays a key role in tumor survival and resistance to treatment [16], could interact with ERK1/2 signaling to amplify the therapeutic effects of MC4R inhibition. Together, these pathways highlight the potential of targeting MC4R to disrupt multiple signaling mechanisms critical for tumor progression.

We treated ATC and colorectal cancer cells with vinorelbine and SN-38 (the active metabolite of irinotecan), the chemotherapeutic drugs conventionally used in clinical practice for the treatment of these two types of tumors [44,45]. The low values confirmed their antitumor activity with experimental IC_50_ values in the picomolar range for vinorelbine in 8305, and in the nanomolar range for HT-29 following treatment with SN-38. These values were obtained with a continuous daily exposure of cells for 72 h to mimic the clinical environment and have proven to be comparable to those already present in the literature [33,34]. Our research group had previously explored the possibility of increasing ML efficacy in combination with a chemotherapeutic agent, like temozolomide or vemurafenib, for melanoma and glioblastoma, respectively, both in vivo and in vitro, and the results supported the need for a combination regime to improve antitumor efficacy while lowering dosage and toxicity. Our present study shows that combining ML with SN-38 and VNR has a synergistic effect in vitro, demonstrating the effectiveness of this strategy in treating all types of cancers. Combining these conventional chemotherapeutic drugs with MC4R inhibition significantly improves their antiproliferative effects, as evidenced by the combination index (CI) values we observed. This synergism is encouraging since it raises the possibility of reducing the dosages of the chemotherapeutic drugs and ML, as shown by DRI values reported in Table 1, that could reduce the adverse reactions of the therapy. Additional support for the synergistic interactions was noted with the Loewe additivity model, which demonstrated that the combination treatments outperform the benefits of the monotherapies and produce a more effective therapeutic approach. The in vitro results were supported and validated by the experiments we performed on HT-29 and 8305C xenograft models. Those in vivo studies revealed that the treatment with ML significantly decreased tumor growth volume in mice without causing significant toxicity, either alone or in combination with vinorelbine or CPT-11.

Crucially, the combination therapies showed greater antitumor effects and resulted in a significant reduction in tumor volume in both tumor types, in comparison to single-agent therapies. Notably, after 8 days of treatment with the ML+CPT-11 combination, a 33% reduction in tumor volume compared to the control group was observed. Similarly, treatment with the ML+VNR combination led to a 42% reduction in tumor volume. These results are consistent with our previous studies, where similar combinatory effects were observed, reinforcing the potential of ML00253764 as a synergistic agent when paired with standard chemotherapeutic drugs.

These results suggest that in a preclinical setting, MC4R inhibition improves the effectiveness of chemotherapy. The combination of VNR and ML00253764 produced comparable antineoplastic effects, with significant differences in mean tumor volume between the treatment and control groups, as shown in Figure 6C. Importantly, there was no need for treatment discontinuation in these experiments (Figure 6D), indicating that the combination therapy was well tolerated and did not induce significant in vivo toxicity. Although long-term toxicity studies have not been conducted, no significant weight loss was observed in the treated mice, suggesting that the combination did not cause excessive toxicity during the treatment period. These findings also support previous studies showing that ML can both reduce and prevent tumor cachexia in mice. Furthermore, previous studies from our group included COMET assays and Cytome assays to assess genotoxicity, providing additional confidence in the safety profile of the treatment.

When administered simultaneously, ML plus VNR, and ML plus CPT-11, both significantly increased the apoptotic index in tumor tissues, in line with the antiproliferative effects observed in vitro. In the 8305C tumor, the combined treatment induced a statistically significant increase in caspase 3 expression, suggesting enhanced apoptosis in the treated samples compared to controls. Furthermore, a notable decrease in CD31 expression was observed, reflecting reduced microvascular density in the treated tumors. These findings indicate that MC4R inhibition, via ML, may alter tumor vasculature, limiting nutrient supply and contributing to tumor suppression. Immunohistochemical analysis of tumor tissue from both HT-29 and 8305C xenografts (Table 2) revealed that, in all treated groups, the activation of caspase-3 was significantly increased, while microvascular density (CD31+ staining) was significantly reduced. These results support the proposed mechanism of MC4R inhibition, where enhanced apoptosis and reduced angiogenesis work together to exert antitumor effects. Notably, MC4R expression was also detected on endothelial cells (HUVECs) in this study, as illustrated by immunoblotting results (Figure 3), suggesting that the receptor could directly contribute to decreased microvascularization within tumors. Taken together, these effects strengthen the therapeutic potential of MC4R inhibition as a strategy for suppressing tumor growth through both pro-apoptotic and anti-angiogenic mechanisms.

In light of the promising results observed in our study, further investigations are warranted to explore the full therapeutic potential of MC4R antagonists. One important avenue for future research is to test the efficacy of MC4R antagonists in combination with immunotherapies, which could significantly enhance treatment outcomes by synergistically targeting both tumor cells and the immune system. Additionally, the role of MC4R inhibition in preventing metastasis remains an intriguing area for exploration, as this could provide new strategies to combat tumor spread and improve overall cancer management. Furthermore, our study highlights the potential of MC4R-targeted therapies to address the unmet needs in the treatment of colorectal and anaplastic thyroid cancers. Given the ability of MC4R inhibition to suppress tumor proliferation and promote apoptosis, particularly in combination with chemotherapeutic agents, this approach could offer a much-needed targeted treatment option for these challenging and often resistant cancer types.

## 5. Conclusions

In conclusion, this study introduces MC4R as a novel target for anticancer therapy in colon-rectal cancer and ATC. Our findings demonstrate that MC4R inhibition induces apoptosis, blocks tumor growth, and synergizes effectively with chemotherapy drugs, highlighting its potential for combination treatments to improve patient outcomes.

Given the limited therapeutic options for ATC and the high recurrence rates in advanced colorectal cancer, targeting MC4R could provide a valuable therapeutic alternative to existing treatments. While these findings provide a strong foundation, the preclinical nature of this study and the need for further validation across different tumor types are key limitations that should be addressed. The relatively small number of human cell lines employed in the experiments may not fully reflect the heterogeneity observed in patients’ tumors, which could limit the generalizability of our findings. Finally, we did not evaluate the long-term safety of MC4R antagonists, which is a critical aspect of translating these findings into clinical applications. We plan to address these limitations in future studies to expand the applicability of our results and strengthen the evidence for MC4R as a therapeutic target. Future research should focus on advancing preclinical trials in other cancer models and designing early-stage clinical trials to assess the safety and efficacy of MC4R antagonists in humans. Additionally, understanding the role of MC4R in modulating the tumor microenvironment, particularly its interactions with immune cells, and exploring its combination with immune checkpoint inhibitors, could open new options for therapy. By focusing on the melanocortin pathway, an underexplored area in oncology, this study provides a novel therapeutic approach with the potential to significantly impact the treatment landscape for these challenging cancers.

## Figures and Tables

**Table 1:** Dose reduction index (DRI) values for the drug combinations at 30, 50, 70, and 90% level of inhibition of HT-29 and 8305C cell growth.

8305C	DRI Values			
Affected Cell Fraction (%)	30	50	70	90
VNR ML	4.5820 65.4630	1.6770 122.6360	0.6130 229.7390	0.1240 624.5620
HT-29	DRI Values			
Affected cell fraction (%)	30	50	70	90
SN-38 ML	74.7580 1.1450	137.5620 2.1280	253.1270 3.9550	668.7820 10.6210

**Table 2:** Immunohistochemistry of HT-29 and 8305C subcutaneous tumor. Values are shown as mean ± sem. * *p* < 0.05 vs. vehicle-treated controls.

HT-29	Control	CPT-11 100 mg/kg	ML 30mg/kg	CPT-11 + ML (100mg/kg + 30mg/kg)
Microvascular count (CD31+)	5.60 ± 0.51	7.25 ± 0.95	4.20 ± 0.37	* 4 ± 0.71
Caspase 3	27% ± 3%	40% ± 4%	48% ± 4%	55% ± 6%
8305C	Control	VNR 10 mg/kg	ML 30 mg/kg	VNR + ML (10 mg/kg + 30 mg/kg)
Microvascular count (CD31+)	6.67 ± 0.80	3.50 ± 0.43	3.83 ± 0.48	3 ± 0.55
Caspase 3	25% ± 7%	38% ± 7%	27% ± 2%	46% ± 7%

## Data Availability

The data presented in this study are available on request from the corresponding author.

## Supplementary Materials

The following supporting information can be downloaded at https://www.mdpi.com/article/10.3390/jcm14041165/s1, Figure S1: (A): Antiproliferative effect of vinorelbine in vitro on human 8305C ATC cell lines. The antiproliferative effects of the drug were studied after 72 h of exposure. The data are presented as mean (±SEM) percentage values of vehicle-treated cell proliferation. * *p* < 0.05 vs. controls. (B): Antiproliferative effect of SN-38 in vitro on human HT-29 colon rectal cell lines. The antiproliferative effects of the drug were studied after 72 h of exposure. The data are presented as mean (±SEM) percentage values of vehicle-treated cell proliferation. * *p* < 0.05 vs. controls.
