# Supplementary material for: Melanocortin-4 Receptor Antagonism Inhibits Colorectal and Anaplastic Thyroid Cancer In Vitro and In Vivo
Source: J Clin Med. 2025 Feb 11;14(4):1165. doi: 10.3390/jcm14041165 (PMC11856147; doi:10.3390/jcm14041165)
Supplement: Supplementary file 1 [file jcm-14-01165-s001.zip › jcm-3403040-supplementary.pdf]

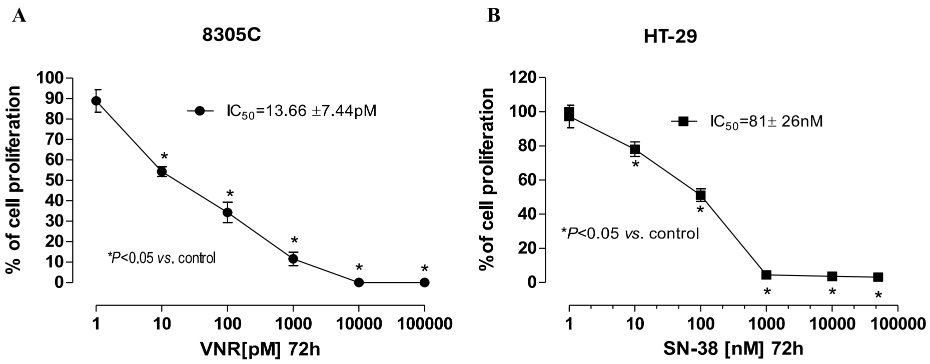

Figure S1. A: Antiproliferative effect of vinorelbine in vitro on human 8305C ATC cell lines. The antiproliferative effects of the drug were studied after 72 h of exposure. The data are presented as mean (±SEM) percentage values of vehicle-treated cell proliferation. * P <0.05 vs. controls. Figure S1B: Antiproliferative effect of SN-38 *in vitro* on human HT-29 colon rectal cell lines. The antiproliferative effects of the drug were studied after 72 h of exposure. The data are presented as mean (±SEM) percentage values of vehicle-treated cell proliferation. * P <0.05 vs. controls.
